# Supplementary material for: Tumor microbiome contributes to an aggressive phenotype in the basal-like subtype of pancreatic cancer
Source: Commun Biol. 2021 Aug 31;4:1019. doi: 10.1038/s42003-021-02557-5 (PMC8408135; doi:10.1038/s42003-021-02557-5)
Supplement: Supplementary file 2 — Supplementary Information [file 42003_2021_2557_MOESM2_ESM.pdf]

Supplementary Figures

Tumor microbiome contributes to an aggressive phenotype in the basal-like subtype of pancreatic cancer

Supplementary Figure 1

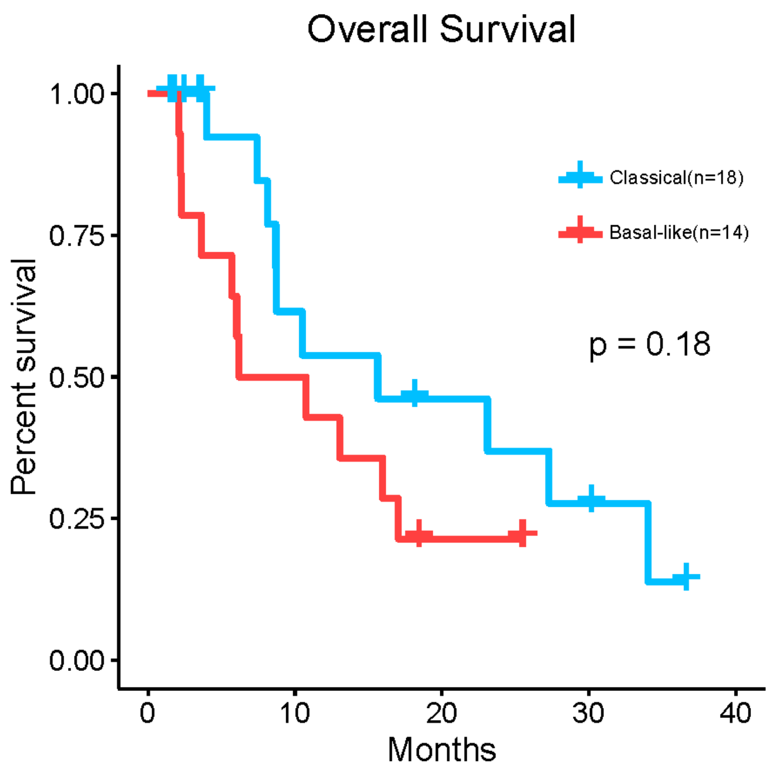

Supplementary Figure 1. Kaplan-Meier survival analysis of different PDAC subtypes.

Kaplan-Meier estimates for the survival probability between basal-like tumors and classical tumors of PDAC. P value is from the log-rank test.

Supplementary Figure 2

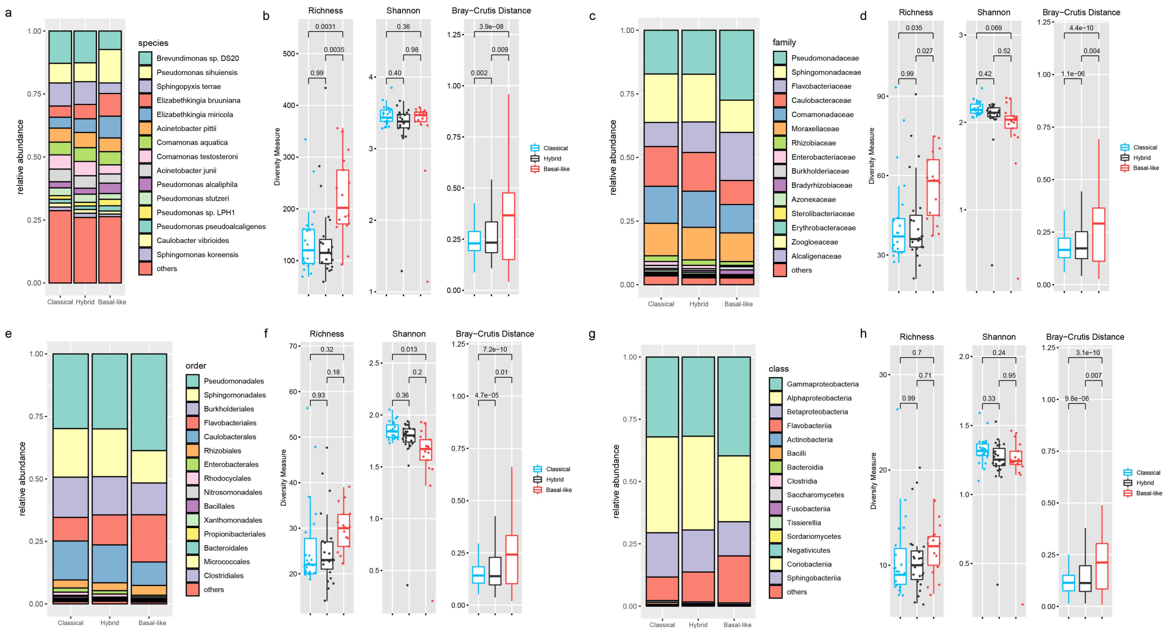

Supplementary Figure 2. Tumor microbiome compositions and diversity measures at various taxonomic levels.

a-h, Barplots displaying the taxonomic composition in the 3 tumor subtypes at the species (**a**), family (**c**), order (**e**), and class levels (**g**). The relative abundance of each taxon is shown. Boxplots of microbial diversity measures at the species (**b**), family (**d**), order (**f**), and class levels (**h**) in the 3 tumor subtypes.

Supplementary Figure 3

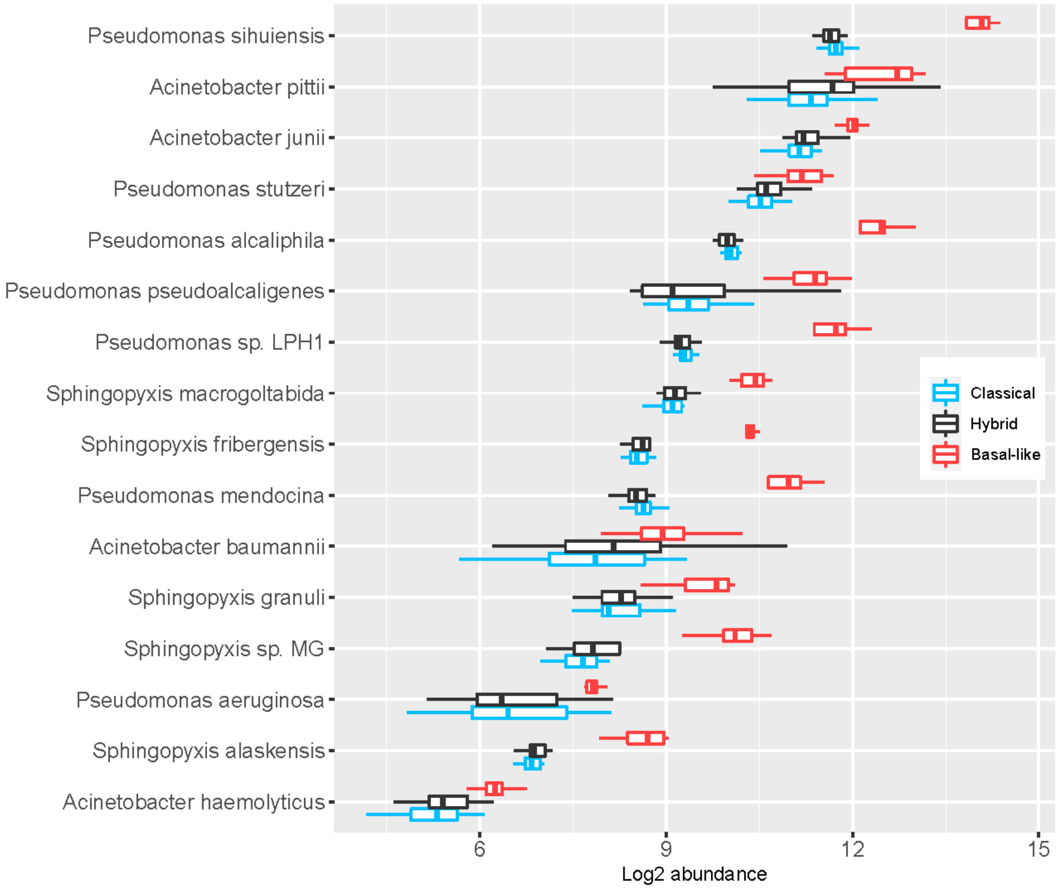

Supplementary Figure 3. Abundance distribution of selected bacteria at species level among PDAC subtypes.

Boxplot showing the abundance of certain bacterial species that belong to the genera of *Acinetobacter*, *Pseudomonas*, and *Sphingopyxis*. The *x* axis indicates the logarithmic value of normalized counts for better visualization. Kruskal-Wallis test was used to determine significance (P value < 0.05).

Supplementary Figure 4

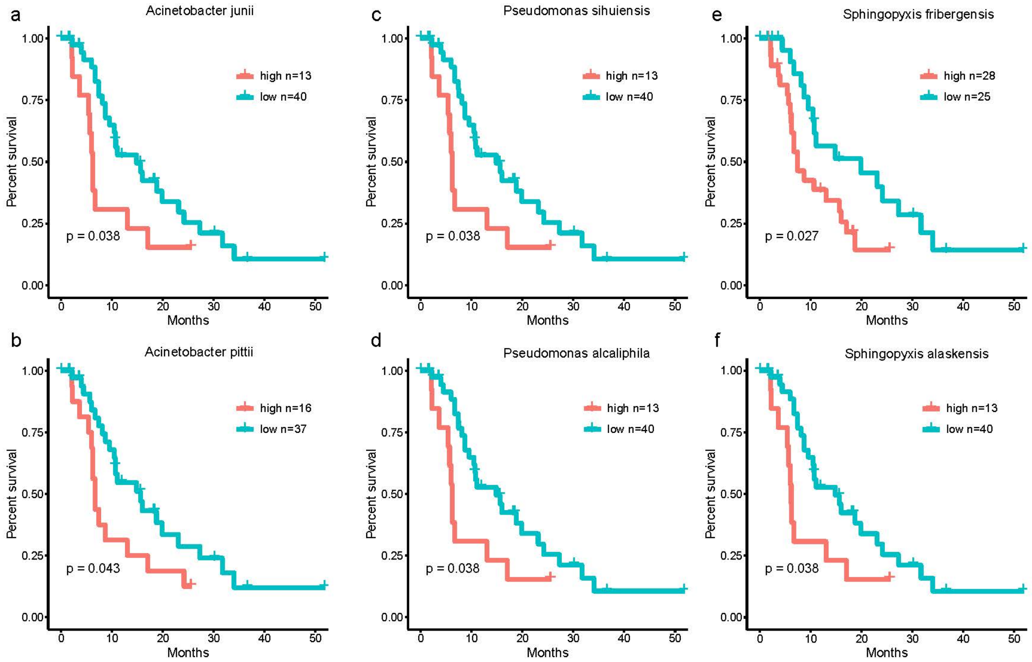

Supplementary Figure 4. Survival analysis based on the abundance levels of specific species.

Kaplan-Meier estimates for survival probability based on the abundance levels of bacterial species *Acinetobacter junii* (a), *Acinetobacter pittii* (b), *Pseudomonas sihuiensis* (c), *Pseudomonas alcaliphila* (d), *Sphingopyxis fribergensis* (e), and *Sphingopyxis alaskensis* (f). The p values are from the log-rank test.

Supplementary Figure 5

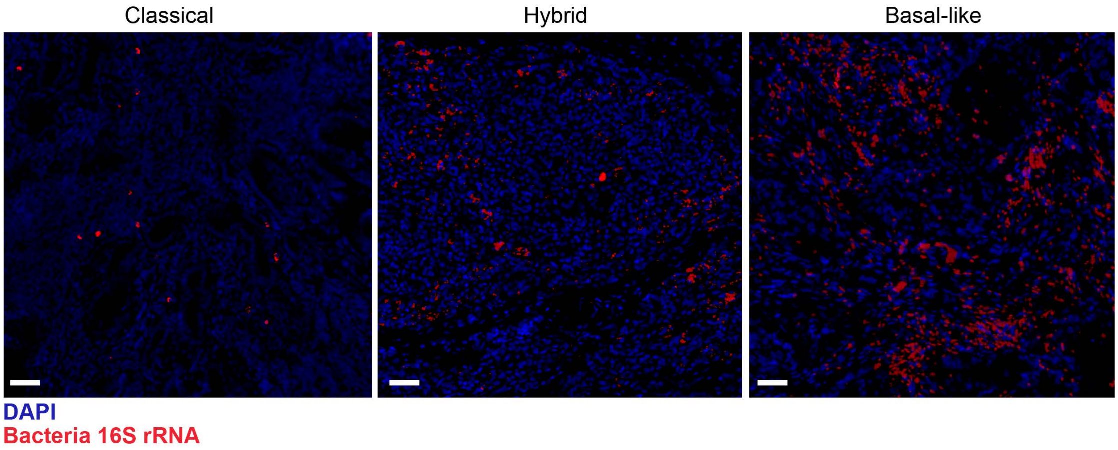

Supplementary Figure 5. Fluorescence in situ hybridization (FISH) in FFPE PDAC tumors.

FISH probes against bacterial 16S rRNA sequences (red) in PDAC samples. Cell nuclei stained with DAPI (blue). The abundance of intrapancreatic bacteria was compared between classical, hybrid and basal-like tumors. Scale bars, 100 µm.

Supplementary Figure 6

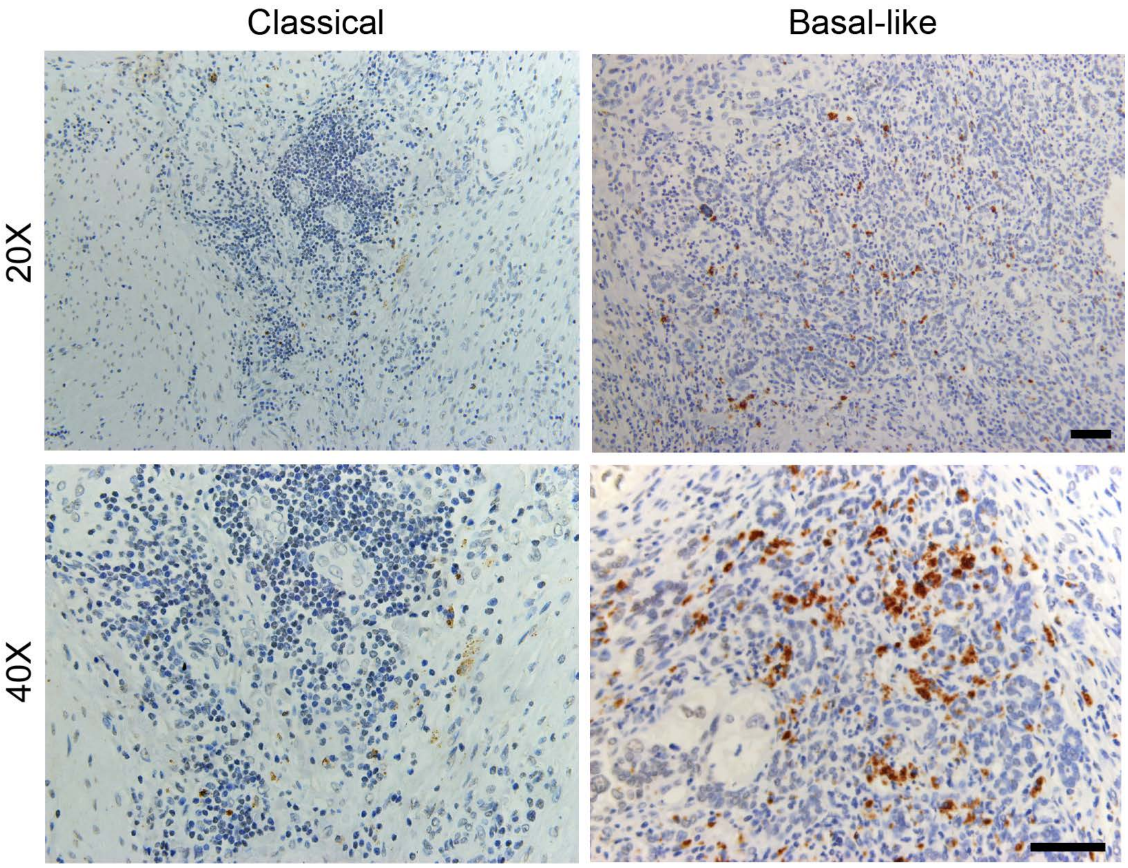

Supplementary Figure 6. Lipopolysaccharide (LPS) staining in FFPE PDAC tumors.

Immunohistochemistry (IHC) using antibodies against lipopolysaccharide (LPS) to detect gram-negative bacteria. Scale bars, 50 μm.

Supplementary Figure 7

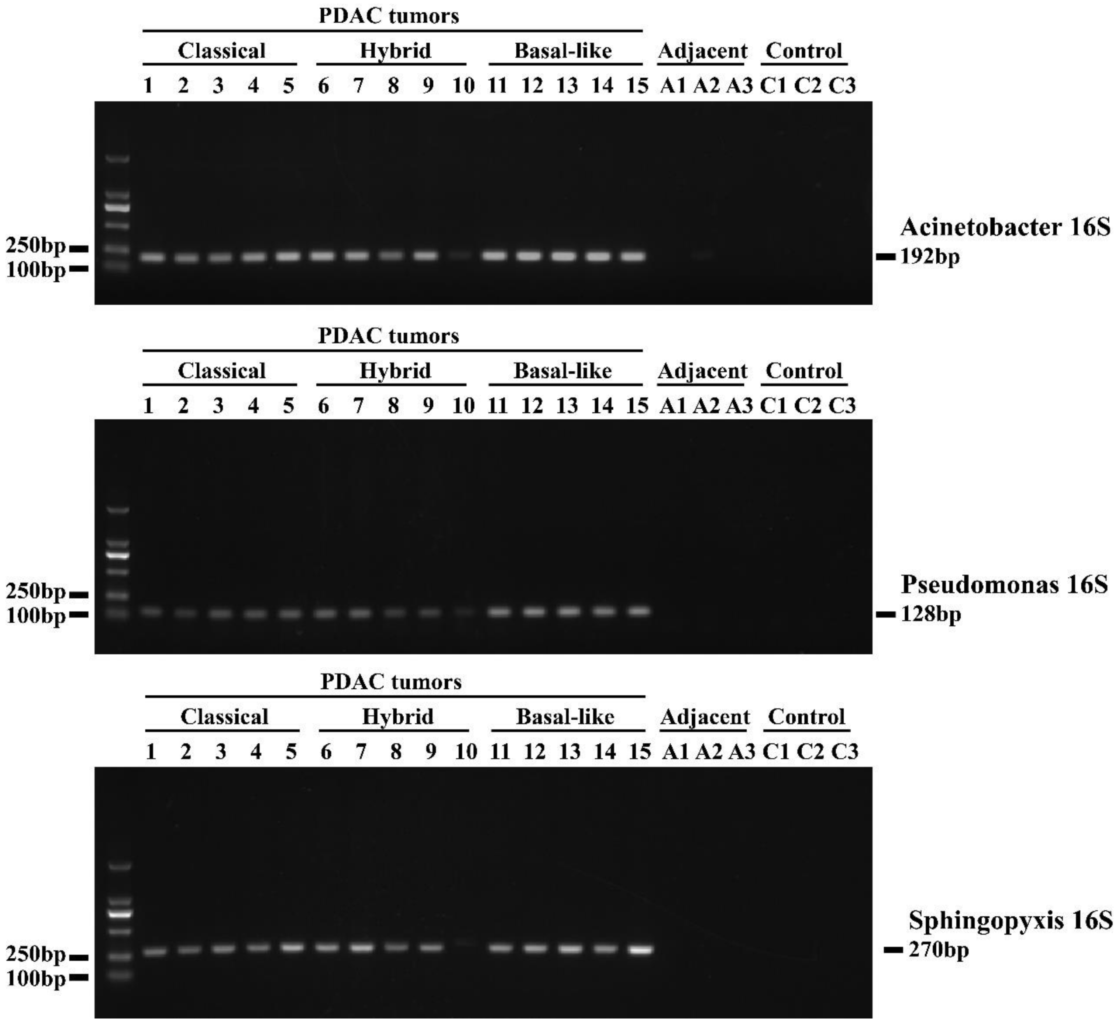

Supplementary Figure 7. PCR validation for bacteria detection.

Genus-specific 16S rDNA PCR executed using primers targeting Acinetobacter, Pseudomonas and Sphingopyxis, shows the presence of bacterial DNA in PDAC tumors, adjacent tissues and several controls. Pancreatic adjacent tissues (A1-A3), environmental sample control (C1), DNA extraction control (C2), and PCR no-template control (C3) were used as negative controls to address contaminants.

Supplementary Figure 8

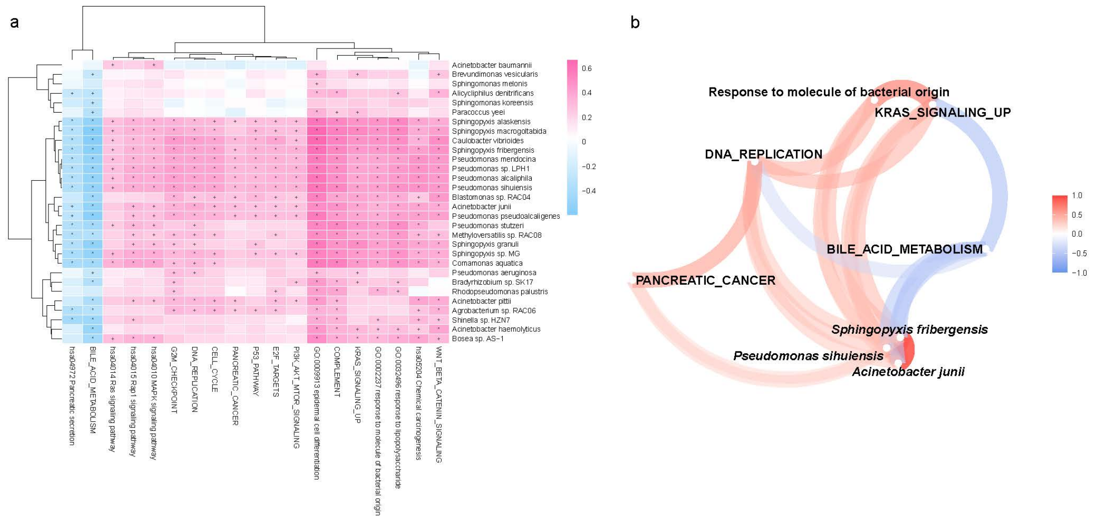

Supplementary Figure 8. The association between host functional modules and the microbial species in PDAC.

a, Heatmap of Pearson's correlation between host functional modules and microbial species enriched in basal-like tumors. The color denotes the coefficient of correlation. + denotes the p values <0.05, and * denotes the p values <0.01. **b,** Correlation network between 5 functional modules and 3 representative bacterial species from genera *Acinetobacter, Pseudomonas and Sphingopyxis*. Red and blue edges denote Pearson's correlation coefficients >0.3 and <-0.3, respectively.

Supplementary Figure 9

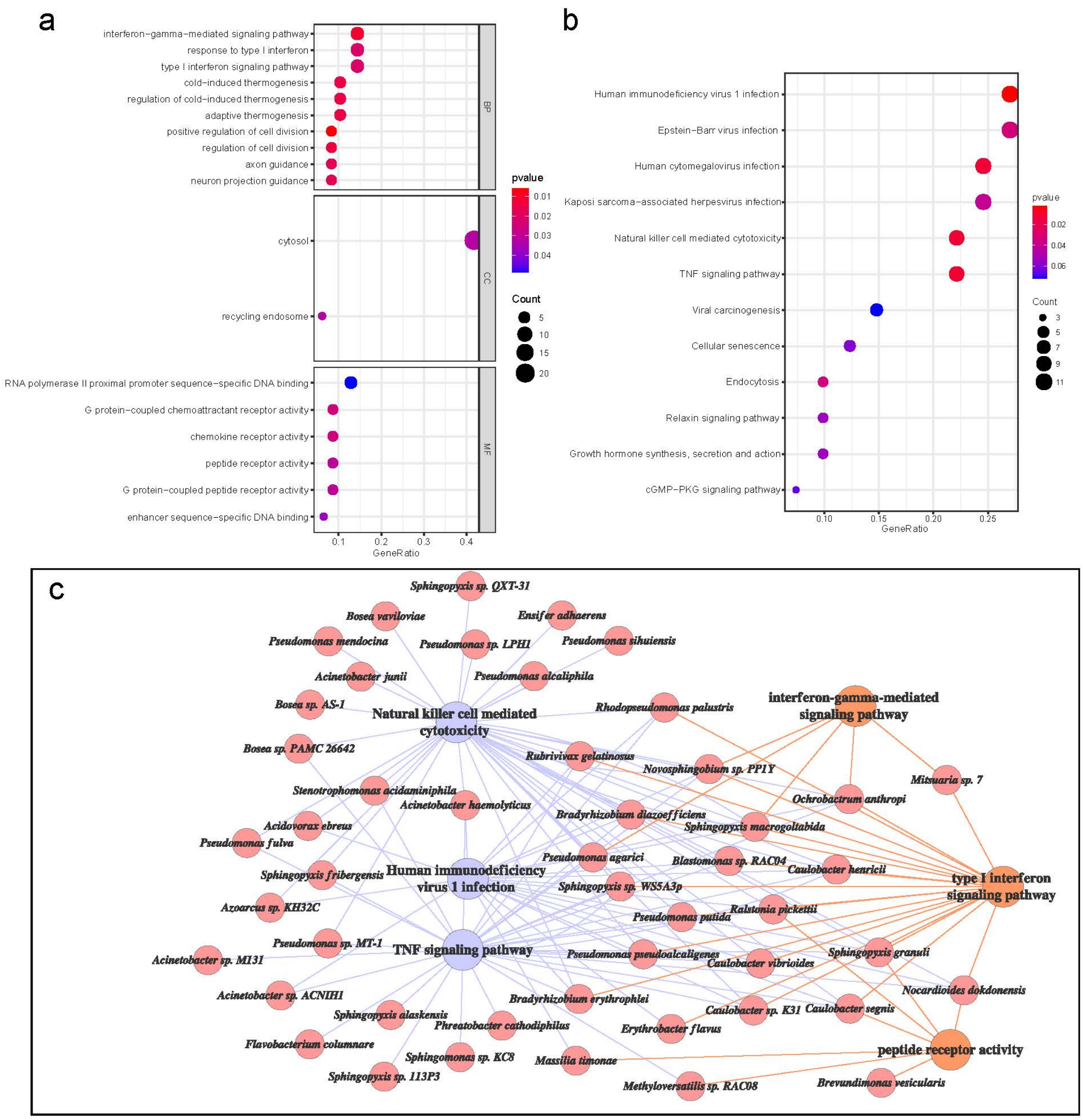

Supplementary Figure 9. Functional enrichment and correlation network of QTLs associated with tumor microbial species.

a, Gene ontology enrichment for genes identified from loci associated with the abundance of tumor microbiome at species level. **b,** KEGG pathway enrichment for genes identified from loci associated with the abundance of species. The color and size of each bubble denote the significance and the number of genes enriched in each functional category, respectively. **c,** Correlation network showing the association between selected functional categories and microbial species. Lines denote the association, and colors represent the types of functions.

Supplementary Figure 10

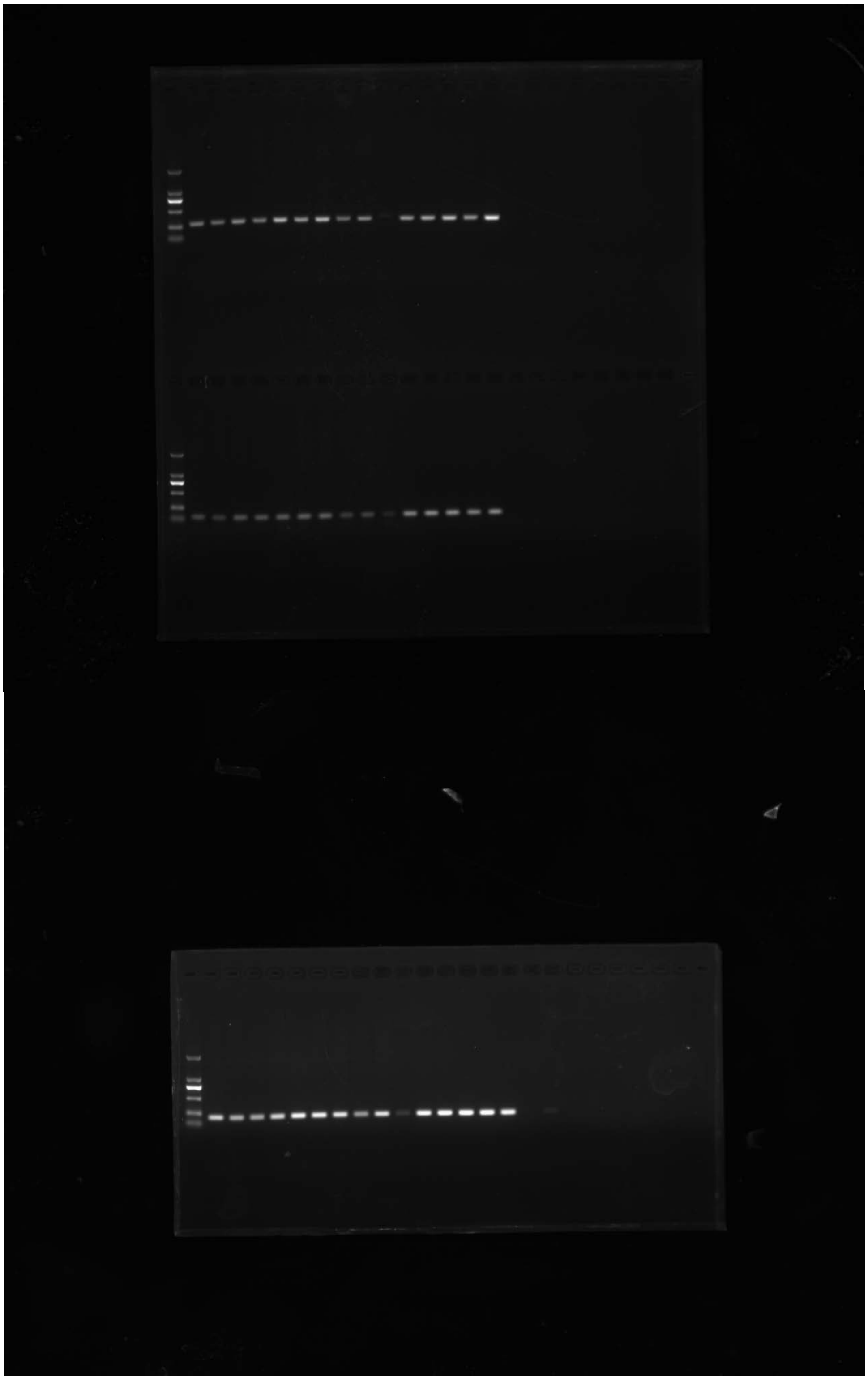

Supplementary Figure 10. Raw images of PCR

Full-sized raw gel images of PCR for Figure S7.
