# Supplementary material for: Tumor microbiome contributes to an aggressive phenotype in the basal-like subtype of pancreatic cancer
Source: Commun Biol. 2021 Aug 31;4:1019. doi: 10.1038/s42003-021-02557-5 (PMC8408135; doi:10.1038/s42003-021-02557-5)
Supplement: Supplementary file 3 — Description of Additional Supplementary Files [file 42003_2021_2557_MOESM3_ESM.pdf]

Description of Additional Supplementary Files

File Name: Supplementary Data 1
Description: Abundance of microbes at each taxonomic level

File Name: Supplementary Data 2
Description: Microbial comparative analysis between tumor subtypes

File Name: Supplementary Data 3
Description: Associations between microbial genera and genetic variations

File Name: Supplementary Data 4
Description: Source data for Figures in the main text
