# Supplementary material for: Tumor microbiome contributes to an aggressive phenotype in the basal-like subtype of pancreatic cancer
Source: Commun Biol. 2021 Aug 31;4:1019. doi: 10.1038/s42003-021-02557-5 (PMC8408135; doi:10.1038/s42003-021-02557-5)
Supplement: Supplementary file 8 — Reporting Summary [file 42003_2021_2557_MOESM8_ESM.pdf]

nature research

Corresponding author(s): Xiangyin Kong

Last updated by author(s): Jul 24, 2021

# Reporting Summary

Nature Research wishes to improve the reproducibility of the work that we publish. This form provides structure for consistency and transparency in reporting. For further information on Nature Research policies, see our Editorial Policies and the Editorial Policy Checklist.

## Statistics

For all statistical analyses, confirm that the following items are present in the figure legend, table legend, main text, or Methods section.

| n/a | Confirmed | |
|---|---|---|
| ☐ | ☒ | The exact sample size (*n*) for each experimental group/condition, given as a discrete number and unit of measurement |
| ☒ | ☐ | A statement on whether measurements were taken from distinct samples or whether the same sample was measured repeatedly |
| ☐ | ☒ | The statistical test(s) used AND whether they are one- or two-sided<br>*Only common tests should be described solely by name; describe more complex techniques in the Methods section.* |
| ☒ | ☐ | A description of all covariates tested |
| ☐ | ☒ | A description of any assumptions or corrections, such as tests of normality and adjustment for multiple comparisons |
| ☐ | ☒ | A full description of the statistical parameters including central tendency (e.g. means) or other basic estimates (e.g. regression coefficient) AND variation (e.g. standard deviation) or associated estimates of uncertainty (e.g. confidence intervals) |
| ☐ | ☒ | For null hypothesis testing, the test statistic (e.g. *F*, *t*, *r*) with confidence intervals, effect sizes, degrees of freedom and *P* value noted<br>*Give P values as exact values whenever suitable.* |
| ☒ | ☐ | For Bayesian analysis, information on the choice of priors and Markov chain Monte Carlo settings |
| ☒ | ☐ | For hierarchical and complex designs, identification of the appropriate level for tests and full reporting of outcomes |
| ☐ | ☒ | Estimates of effect sizes (e.g. Cohen's *d*, Pearson's *r*), indicating how they were calculated |

*Our web collection on statistics for biologists contains articles on many of the points above.*

## Software and code

Policy information about availability of computer code

| | |
|---|---|
| Data collection | No softwares was used in the data collection process. |
| Data analysis | RNA-seq data analysis was performed using HISAT2 (v2.1.0) and HTseq (v0.12.4); count normalization was performed using DESeq2 (v1.24.0); ConsensusClusterPlus (v1.48.0) and pheatmap (v1.0.12) was utilized for clustering; immune cell infiltration was assessed by CIBERSORTX online; WGS sequencing data analysis was performed using Bowtie2 (v2.3.4) and Samtools (v1.8); variant calling was performed using GATK v4.0 pipeline; microbial taxonomic assignment was performed using Kraken2 (v2.0.7) and Bracken (v2.0.0); contigs assembly was performed using MEGAHIT (v1.1.2); gene prediction was performed using MetaGeneMark (v3.38); QTL analysis was performed using MatrixEQTL (v2.2); visualization of correlation was performed by Cytoscape v3.6; further statistical analyses were performed in R (v3.6.3). |

For manuscripts utilizing custom algorithms or software that are central to the research but not yet described in published literature, software must be made available to editors and reviewers. We strongly encourage code deposition in a community repository (e.g. GitHub). See the Nature Research guidelines for submitting code & software for further information.

## Data

Policy information about availability of data

All manuscripts must include a data availability statement. This statement should provide the following information, where applicable:

- Accession codes, unique identifiers, or web links for publicly available datasets
- A list of figures that have associated raw data
- A description of any restrictions on data availability

Raw data are available on NCBI GEO database under accession number GSE172356 and NCBI BioProject under accession number PRJNA719915.

# Field-specific reporting

Please select the one below that is the best fit for your research. If you are not sure, read the appropriate sections before making your selection.

[x] Life sciences [ ] Behavioural & social sciences [ ] Ecological, evolutionary & environmental sciences

For a reference copy of the document with all sections, see nature.com/documents/nr-reporting-summary-flat.pdf

# Life sciences study design

All studies must disclose on these points even when the disclosure is negative.

| | |
|---|---|
| Sample size | 62 RNA-seq; 62 MWGS. |
| Data exclusions | No data were excluded. |
| Replication | No replication cohort was used in this study. |
| Randomization | Not applicable as this study is not a clinical trail. |
| Blinding | Not applicable as this study is not a clinical trail. |

# Reporting for specific materials, systems and methods

We require information from authors about some types of materials, experimental systems and methods used in many studies. Here, indicate whether each material, system or method listed is relevant to your study. If you are not sure if a list item applies to your research, read the appropriate section before selecting a response.

## Materials & experimental systems

| n/a | Involved in the study | |
|---|---|---|
| [ ] | [x] | Antibodies |
| [x] | [ ] | Eukaryotic cell lines |
| [x] | [ ] | Palaeontology and archaeology |
| [x] | [ ] | Animals and other organisms |
| [ ] | [x] | Human research participants |
| [x] | [ ] | Clinical data |
| [x] | [ ] | Dual use research of concern |

## Methods

| n/a | Involved in the study | |
|---|---|---|
| [x] | [ ] | ChIP-seq |
| [x] | [ ] | Flow cytometry |
| [x] | [ ] | MRI-based neuroimaging |

## Antibodies

| | |
|---|---|
| Antibodies used | The mouse monoclonal antibody clone WN1 222-5 recognizes the core region of lipopolysaccharide (LPS) (Lipopolysaccharide Core, mAb WN1 222-5, HycultBiotech, 1:1000 dilution) |
| Validation | As positive control gut or lymph node of SIV-infected rhesus macaques was used and as negative control gut or lymph node of non-SIV-infected rhesus macaques. Estes, J et al; Damaged intestinal epithelial integrity linked to microbial translocation in pathogenic simian immunodeficiency virus infections. PLOS pathogens 2010, 6: e1001052 |

## Human research participants

Policy information about studies involving human research participants

| | |
|---|---|
| Population characteristics | 62 patients with resected tumors of pancreatic ductal adenocarcinoma. |
| Recruitment | Eligible patients required: 1. a radiologic or histologic diagnosis of pancreatic ductal adenocarcinoma; 2. treatment without antibiotic within past one month; 3. surgical resection of tumors. |
| Ethics oversight | Changhai Hospital, Second Military Medical University, Shanghai |

Note that full information on the approval of the study protocol must also be provided in the manuscript.
